# Telocytes promote hepatocellular carcinoma by activating the ERK signaling pathway and miR-942-3p/MMP9 axis

**DOI:** 10.1038/s41420-021-00592-z

**Published:** 2021-08-10

**Authors:** Ying Xu, Hu Tian, Chao Guang Luan, Kai Sun, Peng Jin Bao, Hua Yu Zhang, Nan Zhang

**Affiliations:** 1 Shandong First Medical University and Shandong Academy of Medical Science, Shandong Cancer Hospital and Institute, Ji’nan, Shandong China; 2 The First Affiliated Hospital of Shandong First Medical University, General Surgery, Ji’nan, Shandong China; 3 Ji ‘nan Municipal Three Hospitals, General Surgery, Ji’nan, Shandong China; 4 Affiliated Hospital of Shandong University of Traditional Chinese Medicine, Ji’nan, Shandong China

**Keywords:** Cancer stem cells, Cell invasion

## Abstract

In China, hepatocellular carcinoma (HCC) is considered a malignant tumor with poor prognosis, frequent metastasis, and a high relapse rate. Telocytes (TCs) participate in tumorigenic, invasive, and migratory processes by secreting functional proteins and transmitting cell-to-cell information, but their functions in HCC are still unknown. TC counts and MMP9 expression in liver cancer tissues were measured using immunohistochemistry, western blotting, and RT-PCR. Primary TCs from liver para-cancer tissues were cultured in vitro. To verify the role of TCs in HCC, a metastatic cancer animal model was established using three types of liver cancer cell lines in vivo. TCs promoted HCC cell metastasis by MMP9 expression in vitro and in vivo. Platelet-derived growth factor-alpha (PDGF-α), secreted by HCC cells, activated the Ras/ERK signaling pathway in TCs, thereby increasing MMP9 expression; Moreover, miR-942-3p suppressed MMP9 expression in TCs. Our results reveal the role of TCs in HCC and the mechanisms by which they elicit their effects, and they may serve as novel prognostic markers for HCC.

## Background

Globally, liver cancer is the sixth most common cancer and ranks fourth in terms of cancer-related mortality [1]. The World Health Organization estimates that more than 1 million patients will die of liver cancer by 2030 [2]. Metastasis, a common lethal feature in most malignant carcinomas, which involves secondary germination to distant organs, occurs through multiple steps, such as invasion of the circulatory system after tumor angiogenesis, sustainable growth with inexhaustible viability, and enhanced colonization capacity [3]. Cancer cells can overcome many obstacles by modifying the surrounding environment and regulating peripheral cells to establish appropriate conditions for metastasis. Although various efforts have been made to explore the molecular mechanisms involved in hepatocellular carcinoma (HCC), its prognosis is still made based on the clinical stage, distant metastatic organs, and post-surgical recurrence [4]. There is an urgent need for the discovery of novel biomarkers related HCC metastasis and prognosis, and of new therapeutic targets.

Telocytes (TCs), derived from interstitial Cajal-like stem cells (ICLCs), are a novel type of mesenchymal cell with several long, thin, and bead-like telopodes (Tps), and extensively exist in most mammalian organs [5–9]. TCs are positive for CD34, CD117, and PDGFR-α, and negative for CD28, vimentin, and nitric oxide synthase (NOS) in hepatic, renal, and vascular tissues [10]; these biomarkers are used for TC identification [11–13]. Studies on the role of TCs in tumorigenesis and cancer metastasis have been limited to their morphological and quantitative alterations in tumors. For instance, hyperplastic TCs were identified as the physiological counterparts of inflammatory fibroid polyp neoplasia and PDGFR-α mutant gastrointestinal stromal tumors [14]. Steroid hormone receptors on the membranes of TCs are involved in uterine leiomyoma growth by changing their density and local homeostasis [15]. Therefore, in this study, we sought to explore the potential mechanisms by which TCs influence tumor growth and cancer metastasis.

MMP9, one of the matrix metalloproteinases (MMPs) that participates in matrix remodeling of tissues [16, 17], and in cancer migration, invasion, and tumorigenesis [18, 19], can be secreted by ICLCs such as TCs through homocellular junctions to interact with surrounding cells. It has been reported that downregulating MMP9 expression suppresses HCC metastasis [20, 21]. Several signaling pathways such as the phosphoinositide 3-kinase/protein kinase B/nuclear factor-kappa B (PI3K/AKT/NF-ĸB) signaling pathway and the transforming growth factor-beta (TGF-β)/SMAD) signaling pathway, control MMP9 expression [21–23]. Inhibition of the extracellular signal-regulated kinase (ERK) was found to reduce MMP9 expression, and gain-of-function was found to be reversed by eliminating mitogen-activated protein kinase (MAPK)-related inhibitors in fibrosarcoma [24]. In addition, iron mediates MMP9 expression through the ERK1/2 signaling pathway in head and neck squamous cell carcinomas [25]. The aim of our study was to elucidate the role played by TCs in HCC and the interactions between HCC cells and TCs, and to propose a possible mechanism for the role of TCs in HCC.

## Results

### Diversity and correlation analyses between TCs and MMP9 in HCC

**Fig. 1:**
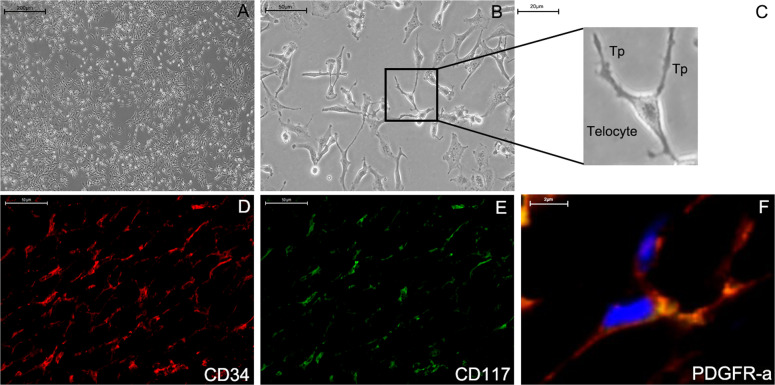
Morphology and immunofluorescent staining of TCs. The unique morphology of TCs was observed by light microscopy (×40 in (**A**); ×200 in (**B**)). The long and thin extending feature is a telopode (Tp, ×400). Three Tps emanating from the body of a TC (**C**). TCs were positive for CD34 (red, **D**), CD117 (green, **E**), and PDGF-α (yellow, **F**).

First, TCs cultured in vitro were authenticated through unique morphological features observed under the light microscopy (Fig. 1A, B) and through CD34-, PDGFR-α-, and CD117-positive immunofluorescence (IF) staining (Fig. 1D–F). The special features of TCs included several long and thin telopodes, which are characteristics that differentiate them from fibrocytes and epithelial cells (Fig. 1C) [26].

**Fig. 2:**
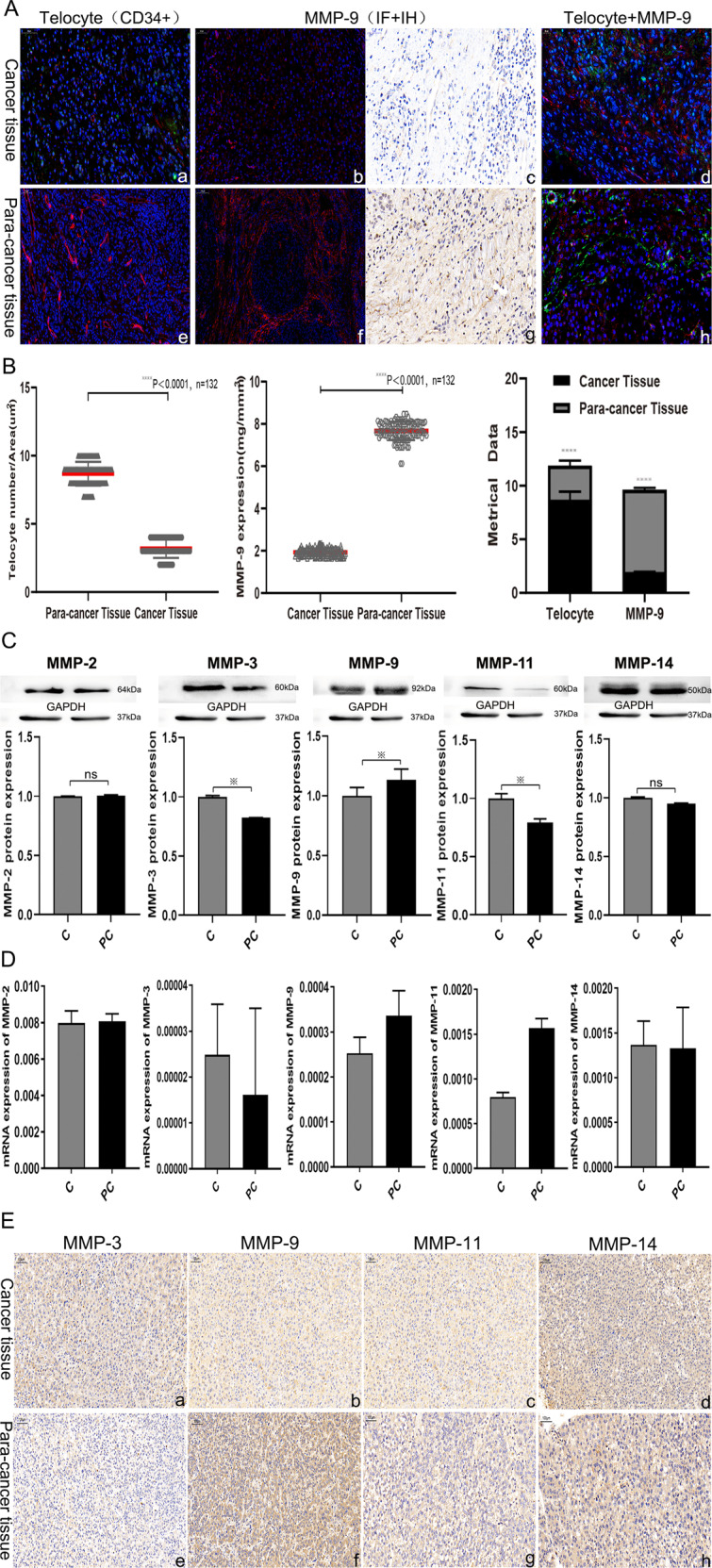
Differences in telocyte and MMP9 expression between the hepatocellular cancer (HCC) and para-cancer tissues. **A** CD34-positive telocytes (red) in the HCC and para-cancer tissue (a, e). MMP9 expression in both tissues as determined by immunohistochemistry and immunofluorescence (b, c, f, g). Distribution of telocytes (CD34+, green) and MMP9 (red) in the same paired tissue, and the relationship between them as determined by immunofluorescence (d, h). **B** Quantified telocyte count and MMP9 expression, and their statistical significance in 132 paired HCC and para-HCC tissue specimen (****p < 0.0001). **C** Some members of the MMP protein family, including MMP2, MMP3, MMP9, MMP11, and MMP14, expressed in 132 HCC tissues as compared to para-HCC tissues as determined by the western blot technique. Glyceraldehyde 3-phosphate dehydrogenase (GAPDH) was used to normalize the concentration. **D** qRT-PCR determination of the distinct expressions of MMP proteins in HCC tissues. **E** Different expressions levels of some MMP proteins in cancer and para-cancer tissues as determined by immunohistochemistry. The star symbol denotes statistical significance at *p* < 0.05. The scale bar denotes 50 μm in (**A**) and 100 μm in (**E**). Data are represented as mean ± SD, and the level of significance was determined using the paired *t*-test.

**Fig. 3:**
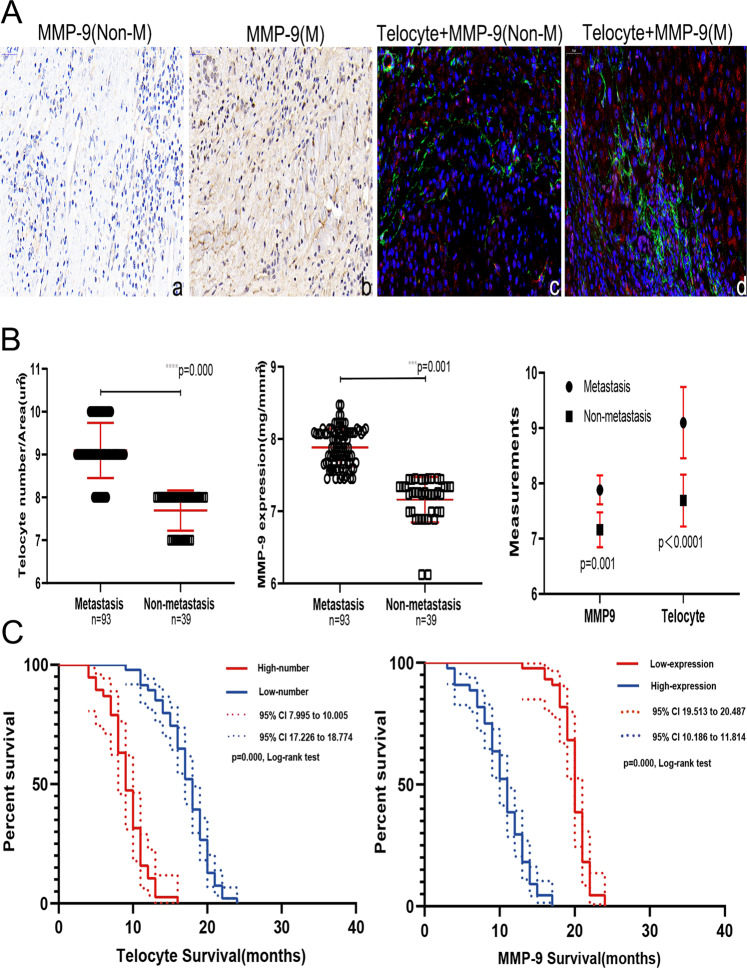
Comparison of telocyte count and MMP9 expression in HCC para-cancer tissues between the metastasis and non-metastasis groups. **A** Different MMP9 expression levels in the HCC tissues without metastasis (a) and with metastasis (b) as determined by immunohistochemistry staining. The relationship and distribution of telocytes (green) and MMP9 (red) in the para-cancer tissues are disparate between non-metastasis (c) and metastasis (d) samples as determined by immunofluorescence staining. **B** Telocytes counts and MMP9 expression in the metastasis (*n* = 93) and non-metastasis (*n* = 39) groups were statistically significantly different. **C** Kaplan–Meier analysis of overall survival (OS) in HCC patients (*n* = 265) with low (red line) or high (blue line) telocyte counts (left panel) and low (red line) or high (blue line) MMP9 levels (right panel) (*p* < 0.05, Log-rank test). ****p < 0.001, ****p = 0.001.

To verify changes in TC counts in HCC tissues, a double IF assay was carried out using 132 specimens to determine the number of CD34+ TCs in HCC and para-cancer tissues (Fig. 2A, panels a, e). TC counts in HCC tissues were significantly lower than those in para-cancer tissues as shown by the paired *t-*test (*t* value = 57.640, *p* < 0.0001, Fig. 2B). MMP9 expression indices in HCC tissues were lower than those in para-cancer tissues (*t* value = 138.600, *p* < 0.0001, Fig. 2A, B). The MMP2, MMP3, MMP9, MMP11, and MMP14 contents of both tissues were determined by western blot analysis, qRT-PCR analysis, and immunohistochemistry (IHC) staining (Fig. 2C, D, E). Expression levels of MMP2 and MMP14 were similar in both tissue types; MMP3 protein expression was lower in para-cancer tissues than in HCC tissues; MMP11 protein expression was higher in HCC tissues. In addition, the correlation between MMP9 expression and TC levels in both tissues was demonstrated through Pearson’s correlation statistical analysis (Table 1). To determine potential clinical correlations among MMP9 expression, TC counts, and HCC metastasis, we analyzed TC counts in tissues obtained from 93 metastasized HCC patients and 39 HCC patients without metastasis. TC counts and MMP9 expression in para-cancer tissues were significantly higher in samples of patients with metastasis than in those of patients without metastasis (Fig. 3A, B). To explore the potential function and significance of TCs in the HCC metastasis process, two hierarchical classifications were established based on TC counts, with low-count being ≤8/μm^2^ and high-count being >8/μm^2^. Patients in the high-TC group were younger than those in the low-TC group (65.510 ± 0.934 vs 75.763 ± 1.640 years, *p* = 0.001). The rate of metastasis was higher in the high-TC group than in the low-TC group (Table 2). Furthermore, MMP9 expression was higher in the high-TC group than it in the low-TC group (7.859 ± 0.349 vs. 7.138 ± 0.273, *p* = 0.001). Overall survival (OS) in the high-TC group was significantly shorter than that in the low-TC group. In the same way, OS was shorter in patients with high MMP9 expression levels than in those with low MMP9 expression levels(high-MMP9 10.455 ± 3.290 vs. Low-MMP9 19.932 ± 2.028 months; Fig. 3C). A multivariate analysis that adjusted for age, sex, metastasis stage, MMP9 expression, and TC count revealed that MMP9 expression and TC count were independent hazard factors for HCC (Table 3).

**Table 1:** Pearson’s correlation of MMP-9 expression and telocyte number in HCC tissues.

		Telocyte number
MMP-9 expression	Correlation coefficient	0.898* 0.687**
	Significance (*p* value)	0.011^a^ 0.001^b^

The analysis implies significant positive correlation between the two factors. The correlation between MMP-9 protein and telocytes is significant.

^a^The cancer tissues (*p < 0.05).

^b^The para-cancer tissues (**p < 0.05).

**Table 2:** Comparison between low and high telocyte groups of HCC patients.

	Low-telocyte (*n* = 38)	High-telocyte (*n* = 94)	*t/p* value
Age, mean ± SD, years	75.763 ± 1.640	65.510 ± 0.934	45.25/0.001
Sex, male/female	16/22	55/39	
Metastasis, number (%)			
M_1_	12 (31.6)	82 (87.2)	
M_0_	26 (68.4)	12 (12.7)	
MMP9, mean ± SD, μm^2^/mg/mm^3^	7.138 ± 0.273	7.859 ± 0.349	13.66/0.001
OS, mean ± SD, months	17.234 ± 3.290	9.263 ± 2.606	13.33/0.000

*MMP9* matrix metalloproteinase-9.

The statistic had significant when *p* < 0.05, MMP9.

**Table 3:** Multivariate analysis of overall survival in HCC patients.

	HR	95% CI	Mutivariate *P*-value
Age	0.633	0.453–0.905	0.077
Sex	0.759	0.536–1.032	0.106
Metastasis	0.402	0.270–0.577	0.000
MMP9 (para-cancer tissue)	0.572	0.480–0.993	0.001
TCs (para-cancer tissue)	0.698	0.412–0.891	0.001

*MMP9* Matrix metalloproteinase-9, *TCs* Telocytes, *HR* hazard ratio, *CI* confidence interval, *HCC* hepatocellular carcinoma.

The statistic had significant when *p* < 0.05.

### Interactions between TCs and HCC cells

**Fig. 4:**
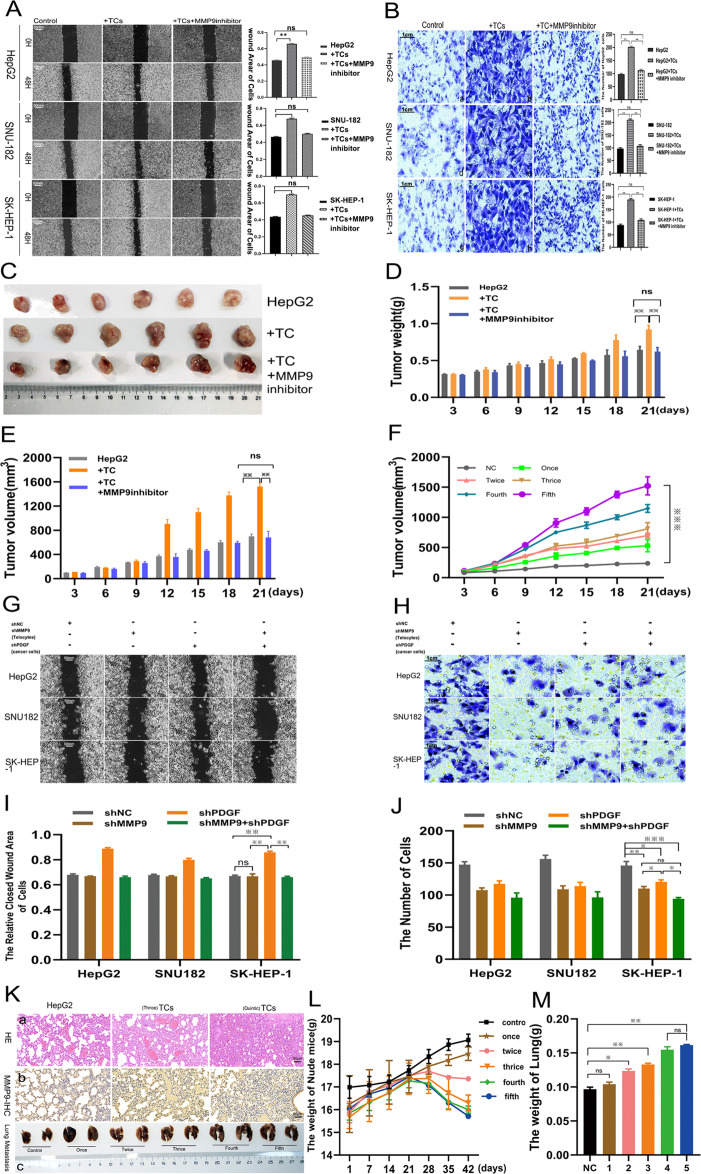
Telocytes promote cancer cell metastasis depending on MMP9 expression. TCs promoted the migration of hepatic cancer cells as determined by the wound healing assay (**A**) and facilitated the invasion of hepatic cancer cells as determined by the Transwell assay (**B**). Representative photos of xenograft tumors in vivo, (**C**) growth curves of tumor weight (**D**) and volume (**E**) over time in the HepG2, TCs, and TCs + MMP9 inhibitor groups. Growth curves of tumor volume with incremental TC injections (**F**). Knockdown of MMP9 and PDGF-α in TCs and HCC, respectively, influenced the migration and invasion of hepatic cancer cells after 48 h as determined by the wound healing and Transwell assays (**G**, **H**). The number of migrating cells (**I**) and invasive cells (**J**) in the shMMP9 and shPDGF-α groups were determined. Representative photographs of metastatic lung tissues derived from HepG2 cancer cells at different frequencies of TCs injection as determined by hematoxylin and eosin staining (**K**-a) and in vivo (**K**-c). MMP9 expression as determined by immunohistochemistry staining in the different groups (**K**-b). Contrast curves of the weights of nude mice (**L**) and the weights of metastatic lung tissues (**M**) at different frequencies of TC injection. ns: no significance; *p < 0.05, **p < 0.01, ***p < 0.005.

To investigate the role played by TCs in HCC, we chose three distinct cancer cell lines on which wound healing and Transwell assays were performed. Our findings indicated that TCs promoted the migration (Fig. 4A) and invasion (Fig. 4B) capacities of HepG2, SNU182, and SK-HEP-1 cells, and that inhibiting MMP9 expression attenuated TC function. We designed in vivo assays using xenograft mouse models by injecting HepG2 cells and TCs into mouse axillae (Fig. 4C). After 21 days, the weights and volumes of the HepG2 tumors following the fifth TC injection were significantly greater in the TC injection group than in the control and MMP9 inhibitor groups (*p* < 0.01; Fig. 4D, E). There was no significant difference in migration and tumor growth between the control group and the MMP9 inhibitor group (*p* > 0.05). These findings indicated that TC activity in HCC depended on MMP9. Within a certain range (from the first to the fifth TC injection), higher TC counts were associated with progressively larger tumor volumes (Fig. 4F). To confirm the molecular mechanisms underlying TC activity, we designed a lentivirus loaded with short hairpin RNA (shRNA) to knockdown MMP9 in TCs and PDGF-α in HCC cells, which could create loss-of-function cell-lines. The migration and invasive capacities of HepG2, SNU182, and SK-HEP-1 cells were retested through the wound healing (Fig. 4G, I) and transwell assays (Fig. 4H, J). Without MMP9, TCs lost the function of promoting cancer cell migration and invasion. We also used nude mice to establish lung metastatic liver cancer lesions and distinguished subgroups according to the different frequencies of mouse tail vein TC injections (Fig. 4K). The extent of lung metastatic cancer lesions increased with increasing frequency of TC injection (Fig. 4K, panel a). IHC staining revealed that MMP9 expression levels were higher in the TC injection group than in the control group (Fig. 4K, panel b). The body weights of mice that received 2 TC injections in the TC injection group were lower than those of mice in the control group and mice that received only 1 TC injection (Fig. 4L). Lung tissue weight increased in the TC injection group as compared to the control group (Fig. 4K, panel c; 4 M).

### Mechanism of TC MMP9 expression

**Fig. 5:**
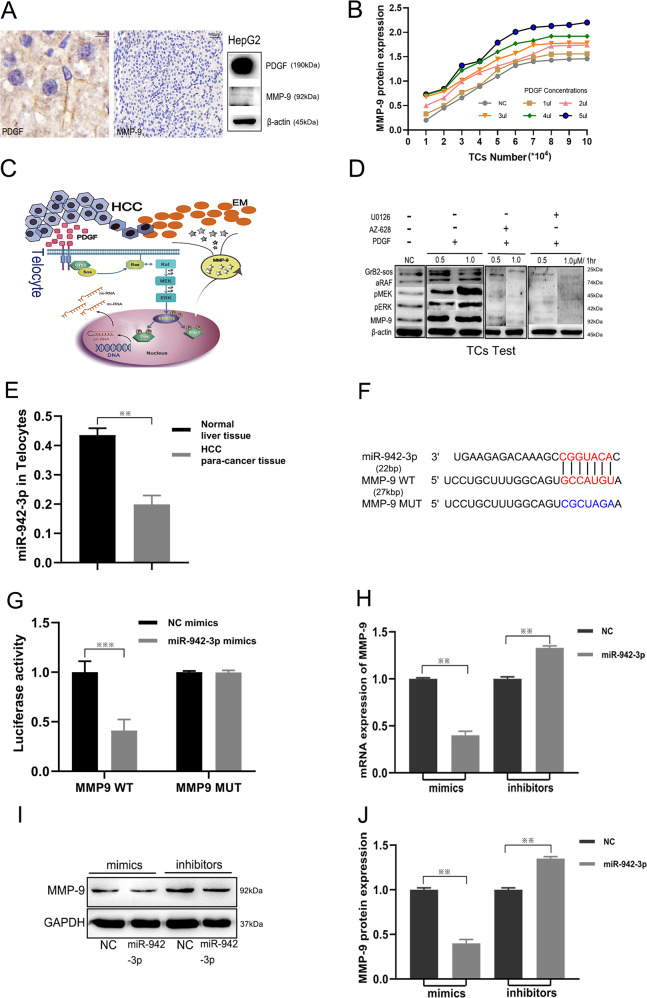
MMP9 expression in telocytes as regulated by the Ras/ERK signaling pathway and miR-942-3p. HepG2 cells secreted PDGF-α, but not MMP9 (**A**). MMP9 expression measured using different TC and PDGF-α concentrations (**B**). Simulation of the mechanism underlying MMP9 secretion by TCs involving the Ras/ERK signaling pathway and relative miRNA (**C**). PDGF-α stimulates TCs to express MMP9 according through the activation of the Ras/ERK signaling pathway that is inhibited by U0126 and AZ-628 (**D**). Differences between the expression levels of miR-942-3p in TCs of HCC para-cancer and normal liver tissues (**E**). Predicted binding site of miR-942-3p on the 5′UTR sequence of MMP9 and mutated sites of MMP9 (**F**). Relative binding observed between miR-942-3p, 5′UTR of MMP9 mRNA, and the mutated MMP9 mRNA as determined by the luciferase reporter gene assay (**G**). Measurement of MMP9 mRNA and protein expression in TCs following overexpression or inhibition of miR-942-3p (**H**, **J**). Differential expression of MMP9 with the administration of miR-942-3p mimics or inhibitors as determined by western blot (**I**).

To clarify the effects of PDGF-α on MMP9 expression in TCs, we first verified whether HCC cells secreted MMP9 using IHF staining, and found that HepG2 cells expressed PDGF-α but not MMP9 (Fig. 5A). MMP9 expression in TCs was determined using different doses of PDGF-α. It was found that 5 μl of PDGF-α acting on 6 × 10^4^ TCs resulted in maximum MMP9 secretion (Fig. 5B). Based on information obtained from the KEGG Database (https://www.genome.jp/kegg/), we found that PDGF-α stimulates TCs to secrete MMP9 by activating the MAPK signaling pathway. We constructed a simulated diagram of the ERK signaling pathway to demonstrate this molecular mechanism (Fig. 5C) [27]. Comparison with the negative control group revealed that PDGFR-α, functioning as a receptor on the membrane of TCs, transmitted an activation signal via Son Of Sevenless (SOS) and growth-factor-bound-2 (Grb2) to stimulate downstream effector proteins for the induction of Ras/ERK signaling. Grb2-sos compound activation, RAF activation, and MEK/ERK phosphorylation, as well as MMP9 expression, were detected by western blot (Fig. 5D). With the administration of the RAF inhibitor, AZ-628 [28], and the ERK inhibitor, U0126 [29], RAF activation, phosphorylation of MEK/ERK, and MMP9 expression were suppressed (Fig. 5D).

According to the miRbase and TargetScan databases, MMP9 is an accurate target of miR-942-3p. Through qRT-PCR analysis, miR-942-3p expression in TCs from HCC para-cancer tissues was found to be higher than that in TCs from normal liver tissue (Fig. 5E). Therefore, a luciferase reporter gene assay was performed, and it was observed that miR-942-3p-mimics significantly reduced MMP9 WT reporter luciferase activity, with no significant change observed in the MMP9 MUT group (Fig. 5F, G). Through qRT-PCR analysis and western blotting, over-expression of miR-942-3p was found to suppress MMP9 expression at both the mRNA and protein levels (*p* < 0.01, Fig. 5H–J).

## Discussion

In this study, multivariate analysis revealed that high TC density and MMP9 expression were correlated with poor OS. These results are consistent with those of previous studies which found an association between abnormal MMP9 expression and breast cancer tumor malignancy [22], lymphatic metastasis, and clinical stage [30], and a close relationship between MMP9 expression and poor primary HCC prognosis [31, 32]. By their basic functions, TCs mainly participate in changing microenvironmental metabolism [33]. Through Spearman’s statistical analysis, we found that MMP9 expression was positively correlated with TC count in HCC para-cancer tissues, and that TCs also secreted MMP9 in vivo. Therefore, we theorized that TCs might produce certain MMPs to regulate tumorigenesis. MMP9 has the capacity to degrade gelatin and type IV, V, and XI collagen, which form the barrier of the ectracellular matrix (ECM) [33] and participate in cancer metastasis [34]. The role of TCs in HCC cell migration and invasion (HepG2, SNU182, and SK-HEP-1 cell lines) was identified in through the transwell and wound healing assays. We found that TCs lost their migration and invasion capacities in the presence of an MMP9 inhibitor in vitro and in vivo. These findings provide the first evidence of how TCs could promote HCC metastasis by producing and secreting MMP9.

HCC cells can secrete multiple chemokines, growth factors, and inflammatory cytokines, including the vascular endothelial growth factor and PDGF-α, to regulate surrounding cells and change the tumor microenvironment (TME). PDGF-α has been associated with lymphangiogenesis and angiogenesis in gliomas, sarcomas, leukemias, and epithelial cancers [35, 36]. PDGF-α and VEGF expression has been associated with poor prognosis in many malignant tumors [37, 38]. HCC cells secrete PDGF-α and VEGF to facilitate cell proliferation, migration, and invasion [39]. In this study, we found that the combination of PDGF-α with PDGFR-α, which is present on the surface of TCs, stimulates TCs to express MMP9. To illustrate the mechanisms involved in the MMP9 related signaling pathway, we used the Kyoto Encyclopedia of Genes and the Genomes database to search for feasible MMP9 signaling pathways. Through this search, we found that PDGF-α affects MMP9 expression via the ERK signaling pathway. Western blot analysis showed that PDGF-α activated GrB2-sos and Raf in TCs, and contributed to MEK1/2 and ERK1/2 phosphorylation, resulting in increasing MMP9 expression. This process could be inhibited using different Raf/ERK inhibitors such as AZ-628 and U0126 as a way to downregulate MMP9 synthesis. We concluded that through MMP9 expression, induced by PDGF-α, TCs promote HCC metastasis by activating the ERK signaling pathway. Therefore, TCs and HCC cells build a network, and TCs can be considered as counterparts of HCC cells as they facilitate cancer cell invasion and migration. Coincidentally, TC hyperplasia, which is manifested by the submucosal thickening, characteristic of the PDGFR-α mutant syndrome, has been pathogenetically associated with inflammatory fibroid polyps and their physiological counterparts in the muscularis propria of gastrointestinal stromal tumors [14]. TCs appear to be crucial in regulating oncogenicity and the neoplastic circumjacent environment. In contrast, a decrease TC count also contributes to genuine fibrosis development in Crohn’s disease, ulcerative colitis, and liver fibrosis [40].

miRNAs are noncoding RNAs constituted of 18–25 nucleotides that degrade mRNA or inhibit translation by binding to the 3′UTR of their target RNAs [41]. Numerous miRNAs directly or indirectly regulate or control cell biological processes, including tumor metastasis and cell division, proliferation, and death. For instance, miR-128-3p functions as a tumor suppressor and triggers cell cycle arrest by repressing LIMK1 expression in breast cancer [42]; downregulation of miR-34a reduces HCC metastasis [31]. Based on TargetScan Database predictions miRNAs associated with MMP9 mRNA in TCs include miR-942-3p, miR-6792, miR-34, and miR-6734. In this study, levels of miR-942-3p in TCs cultured from HCC para-cancer tissues were significantly lower than those in normal liver tissues. Through luciferase and western blot assays, by the decreased cell invasion, migration, and cancer metastasis observed, we demonstrated that miR-942-3p repressed MMP9 expression. Mimics of miR-942-3p significantly reduced MMP9 expression. Downregulation of mi-942-3p was found to be the mechanism of MMP9 toxicity and the springboard used by TCs to promote HCC metastasis.

Two molecular mechanisms underlying the interactions between HCC cells and TCs account for the regulation of MMP9 expression and the mechanism by which TCs accelerate cancer cell metastasis. Activation of the Raf/ERK signaling pathway and downregulation of mi942-3p play pivotal roles in enhancing MMP9 expression and promoting HCC metastasis, respectively. Although miR-942 has been reported to regulate various informational pathways and protein expression, whether it regulated the activation or inhibition of the ERK signaling pathway, or whether PDGF-α can suppress miR-942-3p expression synchronously requires further investigation.

## Conclusion

We demonstrated differences in TC and MMP9 expression in HCC tissues. We also described 2 potential mechanisms by which TCs promote HCC cell metastasis and stimulate MMP9 expression in vitro and in vivo. In addition, we found that PDGF-α, produced by HCC cells, activates the ERK signaling pathway in TCs to stimulate MMP9 expression, and that miR-942-3p suppresses MMP9 expression.

## Materials and methods

### Clinical samples

Between January 2018 and June 2020, surgical tissues were collected at the first affiliated hospital of Shandong first Medical University from 132 patients with HCC confirmed by fast pathology biopsy during the operation, after signed informed consent was obtained from the patients. All fresh tissues were immediately stored in a refrigerator at −80 °C and anonymized before transfer to the laboratory for further processing. Patient demographic data, including age, sex, clinical classification, survival time, and relative follow-up visits were collected. All control subjects, who were free of liver malignancy, were followed up for two and a half years.

### Primary TC culture

Fresh liver para-cancer and hepatic hemangioma (as the control group) tissues, obtained after surgery, were cut into smaller pieces, and incubated with 5 mg/ml collagenase type II (Sigma-Aldrich, St. Louis, MO, USA) for 10 min. Then, samples were washed twice with calcium- and magnesium-free PBS (pH 7.4, G0002, Servicebio, USA) and centrifuged at 10000 r.p.m. for 5 min, and then resuspended in DMEM (Gibco-8120217, NY, USA) supplemented with 10% fetal calf serum. The BJ-40 capillary glass tube (1.0 mm outer diameter, 0.8 mm inner diameter; Hengtong Technology Company, Beijing, China) was soaked in 1 mol/L hydrochloric acid for 24 h, rinsed continuously with ultrapure water, dried at 65 °C, and autoclaved. Under ×200 magnification of the microscope and selected cells to 0.2 mL centrifuge tubes containing 2 μL of lysate according to the morphology of TCs [43, 44].

### Liver TC isolation and identification

Following the sacrifice of male mature C57BL/6 mice (No. 4432, Weitonglihua animal company, Beijing, China), 20-weeks old, using an anesthetic, mouse hepatic tissue was collected under sterile conditions and transferred into sterile tubes containing DMEM supplemented with 100 UI/ml penicillin and 0.1 mg/ml streptomycin (20201013, Kaisu biology Co Ltd., Jiangsu, China), and transported to the cell culture laboratory. Dispersed cells were separated by filtration through a 40 m-diameter cell strainer (CLS431751, Falcon, NJ, Germany), collected by centrifugation at 1000 rpm for 5 min, and resuspended in DMEM supplemented with 10% fetal calf serum, 100 UI/ml penicillin, and 0.1 mg/ml streptomycin (Sigma-Aldrich). Cells were distributed in 25 cm^2^ plastic culture flasks at a density of 1 × 10^5^ cells/cm^2^ and maintained in a 37 °C and 5% CO_2_ atmosphere until they became semi-confluent. Typical TCs were photographed by auto-microscopy every 12 h. After the adhesion of cells to the plate, TCs were selected, purified, and further multiplied for the next experiment. TCs were identified according to their morphology and immunofluorescent staining assays [45].

### Lentivirus production and transfection

To build recombinant lentiviruses, 293T cells were co-transfected with progresses of package, envelop, and expression. The virus-containing supernatant was collected and concentrated by ultracentrifugation. The viral stock was supplemented with 8 mg/mL polybrene for infection.

### Cell transfection

To obtain stable experimental cell lines, primary TCs from mouse hepatic tissues were transfected with SV40 large and small T antigens to obtain TC^SV40^. TC^SV40^ cells were cultured in Dulbecco’s modified Eagle’s medium/F12 supplemented with 10% fetal calf serum (Gibco-8120330, NY, USA). HepG2, SNU182, and SK-HEP-1 cell-Lines were cultured in DMEM (GIBCO, Beijing, China) supplemented with 10% FBS, 100 U/ml of penicillin, and 100 μg/ml streptomycin. These cells were incubated at 37 °C in a humidified atmosphere containing 5% CO_2_. When cells reached 60–80% confluence, positive and stable transfectants were selected for the subsequent experiments.

### RNA extraction and qRT-PCR analysis

To determine MMP2, MMP3, MMP9, MMP11, and MMP14 mRNA expression levels in HCC and para-cancer tissues, qRT-PCR analysis was performed. Total RNA was extracted from tissues using the Trizol reagent (CW0581, Kangweishiji company, China) according to the manufacturer’s protocol [46]. Reverse transcription and cDNA amplification were performed using the SYBR master mix (CW0957, Kangweishiji company, China) according to the manufacturer’s guidelines. β-actin genes were used as internal controls. The primary sequences were shown in Supplementary Table 1. The computational format of mRNA expression = 2^−△△CT^.

### RNA interference

For the miR-942-3p test, complete miRNA sequences were obtained from the miRBase database; shRNAs and mimics of the indicated miRNA were purchased from RiboBio Company (Shanghai and Wuhan, China). Transfection with shRNAs and miRNAs was performed using riboFECT™ CP (RiboBio, Wuhan, China) according to the manufacturer’s instructions.

### Luciferase assay

For the 3′-UTR analysis, cells were co-transfected with a psiCHECK-2-based construct and pre-miR-942-3p or a negative control. The Luciferase assay was conducted using the Luciferase Reporter Assay System (Promega, Madison, WI, USA). The Luciferase miRNA target expression vector (Promega, Madison, WI, USA) was used to construct the reporter vectors, MMP9 wild type (WT), MMP9 mutant (MUT), and negative vector-mimics (NC).

### Western blot analysis

For western blot analysis, samples were thawed and resuspended using a lysis buffer (20% Glycerol, 4% SDS in 100 mM Tris Buffer, pH 6.8). Cell extracts were boiled for 10 min in loading buffer and then centrifuged at 12,000 rpm for 10 min at 4 °C using a microcentrifuge. The immune-reactive bands were colored using an ECL-PLUS/TM (Amersham company, UK). The antibodies are listed in Supplementary Table 2.

### Immunohistochemistry (IHC) staining

Formalin-fixed paraffin-embedded primary tumor and para-cancer tissues were used for IHC analysis. For heat-induced antigen retrieval, slides were soaked in a citric acid buffer and maintained at a sub-boiling temperature for 8 min. Sections were observed using a light microscope (XSP-C204, CIC, China), and were scanned using a laser scanning confocal microscope (Eclipse Ti-E, Nikon, Japan) at a ×40 magnification. Datums were quantified in IHC digital slides using a Leica Aperio positive pixel count algorithm through whole slide analysis (PANNORAMIC DESK/MIDI/250/1000, 3DHISTECH, Hungary). Antibodies are shown in Supplementary Table 2.

### Immunofluorescence (IF) staining

All sections were incubated twice in xylene for 15 min. The sections were dehydrated in two changes of pure ethanol using a dehydrator and were immersed in an EDTA antigen retrieval buffer (pH 8.0, G1206/G1203, Servicebio, USA). After the antibody reaction, photos were taken using a fluorescence microscope (NIKON ECLIPSE C1, Tokyo, Japan) with an imaging system (NIKON DS-U3, Tokyo, Japan). Images were captured at a magnification of 1–400 (Microscope Camera XSP-C204, Olympus Europa GmbH, Hamburg, Germany). Antibodies are listed in Supplementary Table 2.

### Transwell assay

For the invasion assays, transwell migration chambers and Matrigel-coated chambers (Becton Dickinson, Waltham, MA) were used. In summary, 5 × 10^4^ HCC cells were seeded into the upper chamber in a serum-free culture medium. The lower chamber was filled with 5 × 10^4^ TCs in DME medium(Thermo Fisher, HyClone, UT, USA) with 10% FBS(Thermo Fisher Scientific, MA, USA). After 48 h of incubation, cells that crossed the membrane were stained with 1% crystal violet and quantified using a microscope (CKX-51, OLYMPUS company, Japan).

### Wound healing assay

Using a ball marker, horizontal lines were uniformly drawn on the back of a six-well plate. Approximately 2.5 × 10^5^ HCC cells were added to the wells and incubated overnight to reach a fusion rate of 100%. A TC^SV40^ supernatant was prepared after 24 h of culturing and added to the chambers of the plate. MMP9 inhibitor group was a concentration of 3 µM, adding each group of co-culture chambers into the corresponding wells. Each chamber was incubated at 37 °C in a 5% CO_2_ atmosphere for 48 h. The area was estimated using the Image J software.

### In vivo models

To establish a metastatic lung cancer model, 100 μl of PBS containing 1 × 10^7^ HepG2 cells was injected into the tail veins of BALB/c-nu mice (6- weeks old; No. 4272, Weitonglihua animal company, Beijing, China). After that, 50 μl with 0.9% normal saline containing 6 × 10^4^ TCs was injected weekly. The mice were sacrificed on day 42, and their lung tissues were resected, photographed, fixed with 4% paraformaldehyde, and strained with hematoxylin and eosin (HE). For sub-axillary transplantation, HepG2 cells were injected into the right axilla of nude mice, and 50 μl of 0.9% normal saline containing 6 × 10^4^ TCs was injected around local tumors after every 3 days, with the group that received TCs and the MMP9 inhibitor serving as the comparison group. After 28 days, these mice were sacrificed to obtain sub-axillary transplanted tumors, the weights and volumes of which were measured (maximum axis × minimum axis^2^ × 1/2). In order to gain significant results, the number of animals in different groups should keep at least 5, and the dead mice were excluded from the experiment.

### Statistical analyses

SPSS version 20.0 (SPSS Inc., Chicago, IL, USA) was used for the statistical analyses. Quantitative variables were represented as the mean ± standard deviation (SD). Two-tailed Student’s *t*-tests, paired *t*-tests, chi-square tests, and multivariate analyses were used to evaluate the differences between groups. Pearson correlation analysis was used to determine the relationship between MMP9 expression and TC number in HCC tissues. Survival curves were constructed through Kaplan–Meier analyses, and values of *P* < 0.05 were considered statistically significant.

## Supplementary information

supplement tables

## Data Availability

All data and material in our research are valid and veritable.
