# Supplementary material for: Telocytes promote hepatocellular carcinoma by activating the ERK signaling pathway and miR-942-3p/MMP9 axis
Source: Cell Death Discov. 2021 Aug 10;7:209. doi: 10.1038/s41420-021-00592-z (PMC8355302; doi:10.1038/s41420-021-00592-z)
Supplement: Supplementary file 1 — supplement tables [file 41420_2021_592_MOESM1_ESM.docx]

**Supplement Table 1 The primer sequence of qRT-PCR.**

|  | Upstream Primer Sequence | Downstream Primer Sequence |
| --- | --- | --- |
| MMP2 | TGATGGCATCGCTCAGATCC | GGCCTCGTATACCGCATCAA |
| MMP3 | TGAGGACACCAGCATGAACC | ATCACCTCCAGAGTGTCGGA |
| MMP9 | ACCTTCACTCGCGTGTACAG | GGACCACAACTCGTCATCGT |
| MMP11 | CTGGGTGTACGACGGTGAAA | CTTGTTCTTCTCGGGACCCC |
| MMP14 | GGCTGCCTACCGACAAGATT | GGGAGACTCAGGGATCCCTT |
| β-actin | AGACCTGTACGCCAACACAG | CGGACTCGTCATACTCCTGC |
| miR-942-3p | CACAUGGCCGAAACAGAGAAGU | |

β-actin was considered as the internal reference.

**Supplement Table 2 Details of first and second antibodies in IHC and IF**

| Antibody | Species | Number | Company | Dilution | Country |
| --- | --- | --- | --- | --- | --- |
| MMP2 | Rabbit | GB111507 | Servicebio | 1/100 | China |
| MMP3 | Rabbit | GB11131 | Servicebio | 1/100 | China |
| MMP9 | Mouse | GB12132-1 | Servicebio | 1/100 | China |
| MMP11 | Rabbit | YT2794 | Immunoway | 1/100 | UK |
| MMP14 | Rabbit | 13130 | CST | 1/100 | China |
| Bax | Mouse | 2772S | PTG | 1/1000 | China |
| Cleaved-caspase-3 | Rabbit | 9661S | CST | 1/1000 | UK |
| CD34 | Rabbit | AB81289 | Abcam | 1/200 | UK |
| CD117 | Mouse | GB11073 | Abcam | 1/200 | UK |
| PDGFR-α | Rabbit | GB11261 | Abcam | 1/500 | UK |
| GAPDH | Mouse | 60004-1-Ig | PTG | 1/5000 | UK |
| Second-antibody | Anti-Rabbit IgG/  Anti-Mouse IgG | S0001/S0002 | Affinity | 1/5000 | China |
